# Hepatitis B virus nucleocapsid uncoating: biological consequences and regulation by cellular nucleases

**DOI:** 10.1080/22221751.2021.1919034

**Published:** 2021-05-01

**Authors:** Jin Hu, Liudi Tang, Junjun Cheng, Tianlun Zhou, Yuhuan Li, Jinhong Chang, Qiong Zhao, Ju-Tao Guo

**Affiliations:** a Department of Experimental Medicine, Baruch S. Blumberg Institute, Doylestown, PA, USA; b Institute of Medicinal Biotechnology, Chinese Academy of Medical Sciences and Peking Union Medical College, Beijing, People’s Republic of China; c Microbiology and Immunology Graduate Program, Drexel University College of Medicine, Philadelphia, PA, USA

**Keywords:** Hepatitis B virus, nucleocapsid, nucleocapsid uncoating, cccDNA, cyclic GMP-AMP synthase (cGAS), stimulator of interferon genes (STING), TREX1

## Abstract

Upon infection of hepatocyte, Hepatitis B virus (HBV) genomic DNA in nucleocapsid is transported into the nucleus and converted into a covalently closed circular (ccc) DNA to serve as the template for transcription of viral RNAs. Viral DNA in the cytoplasmic progeny nucleocapsid is another resource to fuel cccDNA amplification. Apparently, nucleocapsid disassembly, or viral genomic DNA uncoating, is an essential step for cccDNA synthesis from both *de novo* infection and intracellular amplification pathways, and has a potential to activate DNA sensors and induce an innate immune response in infected hepatocytes. However, where and how the nucleocapsid disassembly occurs is not well understood. The work reported herein showed that the enhanced disassembly of progeny mature nucleocapsids in the cytoplasm supported cccDNA intracellular amplification, but failed to activate the cGAS-STING-mediated innate immune response in hepatocytes. Interestingly, while expression of a cytoplasmic exonuclease TREX1 in human hepatoma cells supporting HBV replication significantly reduced the amounts of cccDNA as well as its precursor, deproteinized relaxed circular (rc) DNA, expression of TREX1 in sodium taurocholate cotransporting polypeptide-expressing human hepatoma cells did not inhibit cccDNA synthesis from *de novo* HBV infection. The results from this cytoplasmic nuclease protection assay imply that the disassembly of progeny mature nucleocapsids and removal of viral DNA polymerase covalently linked to the 5′ end of minus strand of rcDNA take place in the cytoplasm. On the contrary, the disassembly of virion-derived nucleocapsids during *de novo* infection may occur at a different subcellular compartment and possibly *via* distinct mechanisms.

## Introduction

Hepatitis B virus (HBV) is a member of *Hepadnaviridae* family and contains a 3.2 kb, partially double-stranded, relaxed circular (rc) DNA genome, which is replicated *via* the reverse transcription of a RNA pregenome in cytoplasmic nucleocapsid [1]. Upon infection of hepatocyte, HBV nucleocapsid delivers rcDNA genome into the nucleus, where the rcDNA is converted into an episomal cccDNA for transcription of viral RNAs. A viral RNA species longer than full-length of viral genome, designated as pregenomic (pg) RNA, is translated to yield core protein (Cp) and polymerase (Pol). Pol binding of pgRNA at 5′ stem-loop structure (ϵ) initiates the assembly of nucleocapsid. Inside the nucleocapsid, Pol reverse transcribes the pgRNA to produce a single-stranded DNA and then rcDNA. The rcDNA-containing mature nucleocapsids either acquire envelopes and be secreted out of cells as virions or deliver the rcDNA into the nuclei to amplify the pool of cccDNA [2,3]. CCC DNA is stable in hepatocytes and refractory to the current available antiviral agents and had been demonstrated to be the source of virus rebound after the secession of antiviral therapy [4]. Hence, understanding the molecular mechanism of cccDNA metabolism and its regulation by viral and host cellular factors is critical for the development of antiviral drugs to eradicate or functionally inactivate cccDNA and ultimately cure the chronic HBV infection [5,6].

Apparently, nucleocapsid disassembly, or viral genomic DNA uncoating, is an essential step for HBV infection as well as intracellular cccDNA amplification. However, where and how the virion-derived or cytoplasmic progeny nucleocapsids disassemble to release their DNA contents for cccDNA synthesis has not been well understood [7]. In addition, dysregulated uncoating of viral DNA can potentially activate cytoplasmic and/or nuclear DNA sensors and induce an innate immune response in infected hepatocytes [8–10]. It is thus conceivable that nucleocapsid disassembly ought to be delicately controlled by viral and host cellular factors. As a matter of fact, it had been reported recently that alteration of core protein dimer–dimer interaction by either single amino acid substitution (I126A) of Cp or treatment with core protein allosteric modulators (CpAMs) destabilized the cytoplasmic rcDNA-containing nucleocapsids, which resulted in the reduction of total intracellular rcDNA, but increase of cccDNA and its precursor, deproteinized rcDNA (DP-rcDNA) in human hepatoma cells [11,12]. The association of mature nucleocapsid destabilization and increased cccDNA amplification was also demonstrated in an immortalized mouse hepatocyte line (AML12) [13].

In this study, we investigated the biological consequences of CpAM-induced or Cp mutation-enhanced nucleocapsid disassembly and demonstrated that cytoplasmic uncoating of viral rcDNA is unable to activate cGAS-STING pathway in hepatocytes. The distinct sensitivity of *de novo* synthesis and intracellular amplification of cccDNA to a cytoplasmic nuclease favours the notion that the uncoating of HBV rcDNA from virion-derived nucleocapsids during *de novo* infection and the cytoplasmic progeny mature nucleocapsids during intracellular cccDNA amplification may take place at different subcellular compartments and possibly *via* distinct mechanisms.

## Materials and methods

### Cell culture

Human hepatoblastoma cell line HepG2 was obtained from ATCC. HepAD38 was obtained from Dr. Christoph Seeger at Fox Chase Cancer Center, Philadelphia, USA [14]. HepG2 and HepAD38 derived cell lines with reconstituted human cGAS and STING (HepG2/cGAS-STING and HepAD38/cGAS-STING) or cGAS and mutant human STING with deletion of 39 amino acid residues from carboxyl terminus (HepG2/cGAS-STINGΔC and HepAD38/cGAS-STINGΔC) were described previously [8,15]. Lenti-X 293T and 293A cells were purchased from Clontech. The C3A^hNTCP^ cell line, derived from C3A, a subclone of HepG2 (ATCC HB-8065), and stably expressing human sodium taurocholate cotransporting polypeptide (NTCP) [8]. PXB-cells, the human hepatocytes prepared from chimeric mice (PXB mice) with humanized livers that are highly repopulated by human hepatocytes, were purchased from PhoenixBio (Higashi-Hiroshima, Japan), and cultured with Advanced DMEM/F-12 (Life Tech, Cat. No. 12634-010) supplemented with 2% FBS, SingleQuots (Lonza, Cat. No. CC-4175), 2% DMSO, 2 mM glutamine, 100 U/ml penicillin and 100 µg/ml streptomycin.

### Construction of recombinant adenoviruses

Joint PCR approach was used to introduce I126A into wild type core protein ORF with forward and reverse primers of 5′ GA GTG TGG GCT CGC ACT CCT CCA GCT TAT A 3′ and 5′ AGT GCG AGC CCA CAC TCC GAA AGA CAC C 3′. The resulted PCR fragment was cloned into a parental vector pEN-tetHBV-dS. The pEN-tetHBV-dS plasmid contained a 1.1-unit HBV replicon (genotype D, Genbank accession number V01460) driven by the tet-CMV promoter. pEN-tetHBV-dS did not express any viral envelope proteins due to the presence of a premature stop codon TAA in the S ORF between nts 197–199. The HBV replicon was followed by a cassette of tetracycline transcriptional activator (tTA) whose expression was controlled by CMV-IE promoter. Both the HBV replicon and tTA sequence were recombined into pL-DEST based on the instruction of ViralPower Adenoviral Expression System (Invitrogen, Carlsbad, CA 92008 USA). pL-DEST DNA was next digested with PacI and transfected into 293A cells. One to two weeks post transfection, lysates containing adenovirus was harvested and stored in −80°C for future use.

### Establishment of cell lines expressing wild-type and mutant TREX1

cDNA clones encoding full-length TREX1 and TREX1-D18N were gifts from Judy Lieberman (Addgene Cat. N. 27219 and 27220). The coding sequence of TREX1 was amplified with primers CGCGGATCCATGGGCTCGCAGGC and ATAAGAATGCGGCCGCCTACTCCCCAGGTGT. The resulting PCR product was restricted with BamHI and NotI and inserted into BamHI and NotI-restricted Pcx4bsr vector (Osaka Bioscience Institute, Osaka, Japan). C3A^hNTCP^ and HepAD38 cells were infected with the pseudotyped retroviruses and selected with medium containing 10 μg/ml of Blasticidin. Blasticidin-resistant cells were expanded into cell lines. Proper expression of TREX1 was confirmed by Western blot assays and immunofluorescent assays.

### HBV DNA extraction and Southern blot hybridization analysis

Cytoplasmic core DNA from HBV replicating cell lines or recombinant adenovirus infected cells were extracted as described previously. Protein-free HBV DNA species were extracted with a modified Hirt DNA extraction procedure [16,17]. Both Core DNA and Hirt DNA were resolved in 1.5% agarose gel electrophoresis, transferred onto Hybond-XL membrane and hybridized with an *α*-32P-UTP-labeled minus strand specific full-length HBV riboprobe [18].

### RNA extraction and qRT-PCR analysis of viral pgRNA and cytokine mRNA

Total cellular RNA was extracted with TRIzol reagent by following the manufacturer’s direction (Invitrogen). cDNA was synthesized by using SuperScript III Platinum one-step qRT-PCR Kit (Invitrogen). Real-time PCR assays were performed using a LightCycler 480 II (Roche) with primers reported previously [8].

### Capsid purification and endogenous DNA polymerase assay

HepG2 cells infected with recombinant adenoviruses were cultured with or without 2 mM phosphonoformic acid (PFA) for 6 days. Purification of cytoplasmic capsids and endogenous DNA polymerase assay were performed as previously described [12].

### Western blot assay

Cells grown in a 12-well plate were lysed with 125 µl NuPAGE® LDS sample buffer supplemented with 2.5% 2-Mercaptoethanol. Cell lysates were subjected to heat denaturing at 100°C, loaded on NuPAGE4-12% Bis-Tris Gel and run with NuPAGE MOPS-SDS Running Buffer. Proteins were transferred from the gel onto a PVDF membrane using iBlot 2 Dry Blotting System. Membranes were blocked with TBST (50 mM Tris-HCl, pH 7.6; 150 mM NaCl and 0.1% Tween 20) containing 5% nonfat milk for 1 h and incubated with desired antibody overnight at 4°C. After washing with TBST, the membrane was incubated with LI-COR® IRDye® secondary antibodies. Membranes were washed again with TBST and imaged with LI-COR Odyssey system.

### Immunofluorescent staining

HepAD38 or C3A^hNTCP^ cells and their TREX1 derived cell lines were fixed with PBS containing 4% paraformaldehyde, followed by incubation with 0.1% Triton X-100 for 20 min. The cells were then blocked with PBS containing 1% BSA and 0.1% Triton™ X-100 for 30 min at room temperature. Next, cells were incubated with antibodies against TREX1 for 2 h at room temperature (Santa Cruz). Bound primary antibody was visualized by using Alexa Fluor 594-conjugated secondary antibodies. Cell nuclei were stained with DAPI.

### HBV infection

For HBV infection, C3A^hNTCP^ cells were seeded into collagen-coated 12-well plates at a density of 1.5 × 10^6^ cells per well and cultured in complete DMEM medium containing 3% dimethyl sulfoxide (DMSO). One day later, the cells were infected with HBV prepared from HepAD38 cell culture media at 250 genome equivalents per cell in DMEM containing 4% PEG-8000. The inoculums were removed at 24 h and cells were washed three times with PBS. The infected cultures were maintained in complete DMEM medium containing 3% DMSO until harvesting.

## Results

### Establishment of recombinant adenoviruses supporting tetracycline inducible HBV replication

**Figure 1.**
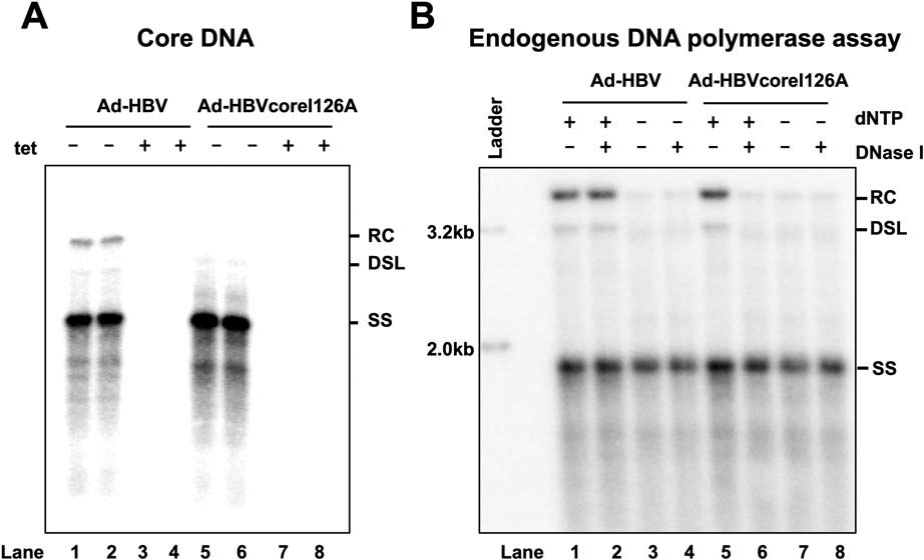
HBV core protein I126A mutation facilitates mature nucleocapsid uncoating. Note: (A) HepG2 cells were infected with Ad-HBV or Ad-HBV I126A at an MOI of 10 and cultured in the absence or presence of 1μg/mL tetracycline (tet). HBV core DNA was extracted at day 6 post infection (dpi) and analysed by Southern blot hybridization. (B) HepG2 cells were infected with Ad-HBV or Ad-HBV I126A at an MOI of 10. The cells were treated with 2 mM PFA from day 2 to day 6 post infection. Cells were then harvested and cytoplasmic HBV capsids were purified by sucrose gradient centrifugation. After endogenous DNA polymerase reaction, capsid DNA were extracted with or without prior DNase I digestion and analysed by Southern blot hybridization. RC, relaxed circular DNA; DSL, double-stranded linear DNA; SS, single-stranded DNA.

In order to investigate the biological consequences and regulation of HBV nucleocapsid disassembly by viral and host cellular factors, we intended to establish an experimental system that can support inducible HBV replication and cccDNA synthesis in different cell lines or primary human hepatocytes. To achieve this goal, we constructed a recombinant adenovirus that expresses HBV pgRNA under a tetracycline (tet) inducible promoter. To abolish the ability of the cells infected by the recombinant adenoviruses to produce HBV virions, a stop codon was introduced into the envelope gene immediately downstream of the starting codon of small envelope protein. Recombinant adenoviruses expressing wild-type HBV pgRNA, designated as Ad-HBV, or pgRNA encoding Cp with I126A substitution, designated as Ad-HBVcoreI126A, were rescued from 293A cells. As shown in Figure 1(A), infection of HepG2 cells by Ad-HBV or Ad-HBVcoreI126A initiated efficient HBV DNA replication when cultured in the absence of tet. However, in agreement with previous reports, examination of viral DNA replication intermediates indicated that expression of I126A mutant Cp resulted in the reduced amounts of mature forms of double-stranded viral DNA, such as double stranded linear (dsl) and relaxed circular (rc) DNA, but did not alter the amount of single-strand (ss) DNA [11] (Figure 1(A)). To distinguish whether the reduced amounts of dslDNA and rcDNA is due to the inhibition of DNA synthesis or degradation of viral DNA by host cellular nucleases upon nucleocapsid disassembly, HBV capsids were purified from the cytoplasmic fraction of the cells treated with a reversible HBV DNA polymerase inhibitor phosphonoformic acid (PFA) to arrest viral DNA replication at the negative strand [19]. An endogenous DNA polymerase assay with cytoplasmic capsids purified from the cells was performed *in vitro* in the absence or presence of dNTP to allow the synthesis of the viral positive strand DNA [12]. To probe the integrity of nucleocapsids, the accessibility of viral DNA to DNase I digestion was tested after the completion of endogenous DNA polymerase reaction, but before the extraction of viral DNA. Southern blot analysis of viral DNA species indicated that rcDNA can be efficiently synthesized in the endogenous DNA polymerase reaction in both wild-type (Figure 1(B), lane 1) and CpI126A mutant (Figure 1(B), lane 5) nucleocapsids when dNTPs were provided. However, while the rcDNA synthesized in WT nucleocapsids was resistant to DNase I (Figure 1(B), lane 2), the rcDNA synthesized in CpI126A mutant nucleocapsids was susceptible to DNase I digestion (Figure 1(B), Lane 6). As anticipated, rcDNA were not synthesized in both WT and CpI126A mutant nucleocapsids in the absence of dNTP (Figure 1(B), lanes 3, 4, 7 and 8). These results imply that the CpI126A mutation enhanced the disassembly of mature nucleocapsids in the cytoplasm, which resulted in the uncoating of viral double-stranded DNA for cccDNA synthesis or digestion by cellular nucleases.

### Enhanced mature nucleocapsid disassembly does not activate cGAS-STING pathway in human hepatoma cells

**Figure 2.**
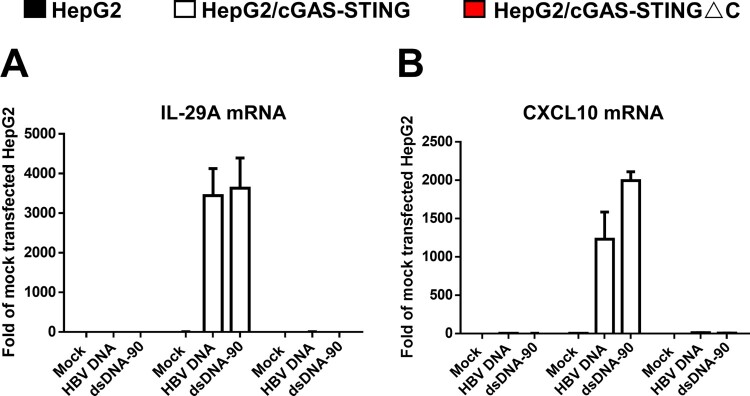
HBV core DNA can activate cGAS-STING pathway upon transfection into human hepatoma cells reconstituted with functional cGAS and STING. Note: Parental HepG2, HepG2/cGAS-STING and HepG2/cGAS-STINGΔC cells were mock-transfected or transfected with 1 μg HBV core DNA or dsDNA-90 per well of 12-well plates and harvested at 8 h post transfection. The levels of IL-29 (A) and CXCL10 (B) mRNA were determined by qRT-PCR assays, normalized to *β*-actin mRNA and presented as the fold of induction over that in mock-transfected HepG2 cells. Mean and standard deviations are presented (*n* = 3).

It is well documented that HBV infection of hepatocytes does not activate cytoplasmic DNA sensors and fails to induce an innate cytokine response [20,21]. While a study demonstrated that hepatocytes are deficient in DNA sensing pathways [22], it is also possible that HBV DNA is not a good ligand for DNA sensors, such as cGAS. To test this hypothesis, we first reconstituted a functional cGAS-STING pathway in human hepatoma (HepG2) cells by expressing human cGAS and STING. As a negative control, a HepG2 cell line expressing wild-type cGAS, but carboxyl-terminally truncated STING was also established [8]. As shown in Figure 2, transfection of double-strand DNA 90 (dsDNA-90), a commercially available cGAS ligand, or HBV core DNA extracted from purified cytoplasmic capsids from HepAD38 cells, can activate the expression of IL-29 and CXCL10 mRNA only in HepG2/cGAS-STING cells, but not in parental HepG2 or HepG2/cGAS-STINGΔC cells. This result is consistent with recent reports suggesting that HBV DNA is competent in activating cGAS-STING pathway [10,23].

**Figure 3.**
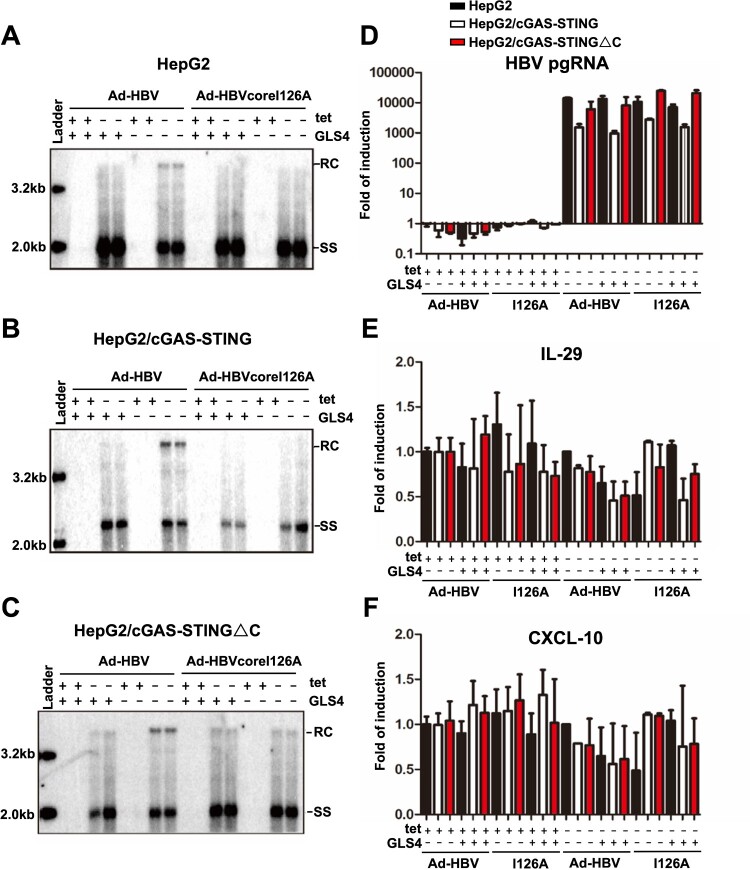
Uncoating of mature HBV nucleocapsids did not activate cGAS-STING pathway. Note: Parental HepG2, HepG2/cGAS-STING, HepG2/cGAS-STING△C cells were infected with Ad-HBV or Ad-HBV I126A at an MOI of 10 and culture in the absence or presence of tet. At day 5 post infection, the cells were mock-treated or treated with 1 μM of GLS4 for 24 h. Cytoplasmic core DNA were extracted and analysed by Southern blot hybridization (Panels (A)–(C)). Total cellular RNA were extracted and the levels of HBV pgRNA (D), IL-29 (E) and CXCL10 (F) mRNA were determined by qRT-PCR assays, normalized to *β*-actin mRNA and presented as the fold of induction over that in mock-transfected HepG2 cells cultured in the presence of tet. Mean and standard deviations are presented (*n* = 3). Black, white and red bars denote parental HepG2, HepG2/cGAS-STING, and HepG2/cGAS-STING△C cells, respectively.

Alternatively, the failure of HBV to activate innate immune response in hepatocytes could also because the viral DNA are shielded by nucleocapsids in the cytoplasm [8]. This hypothesis predicts that the enhanced uncoating of viral DNA in the cytoplasm by treatment with GLS4, a type I CpAM [12,24], or under the condition of CpI126A mutant virus replication may make viral double-stranded DNA accessible to the cytoplasmic DNA sensors and activate a proinflammatory cytokine response. To test this hypothesis, parental HepG2, HepG2/cGAS-STING and HepG2/cGAS-STINGΔC cells were infected by Ad-HBV or Ad-HBVcoreI126A. The infected cells were cultured in the absence or presence of tet for 5 days. The cells were then mock-treated or treated with GLS4 for 24 h. Although HBV pgRNA and DNA levels are slightly reduced in HepG2/cGAS-STING cells (Figure 3(B,D)), CpI126A mutation or GLS4 treatment efficiently induced the reduction of HBV rcDNA in all the three cell lines (Figure 3(A–C)), indicating successful uncoating of double-stranded viral DNA did occur. However, neither IL-29 nor CXCL10 mRNA expression was induced under this condition in all the three cell lines (Figure 3(E,F)). These results thus indicate that although HBV DNA is competent to activate cGAS-STING pathway (Figure 2), the enhanced mature nucleocapsid disassembly in the cytoplasm by GLS4 treatment or CpI126A mutation is insufficient to activate this DNA sensor pathway in human hepatoma cells (Figure 3). Considering the susceptibility of uncoated viral DNA to cellular nucleases (Figures 1 and 3), the possibility that the uncoated viral DNA under these experimental conditions are rapidly digested by unknown cellular nuclease(s) cannot be ruled out.

### Accelerated double-stranded DNA uncoating supports intracellular amplification of cccDNA in human hepatoma cells and primary human hepatocytes

**Figure 4.**
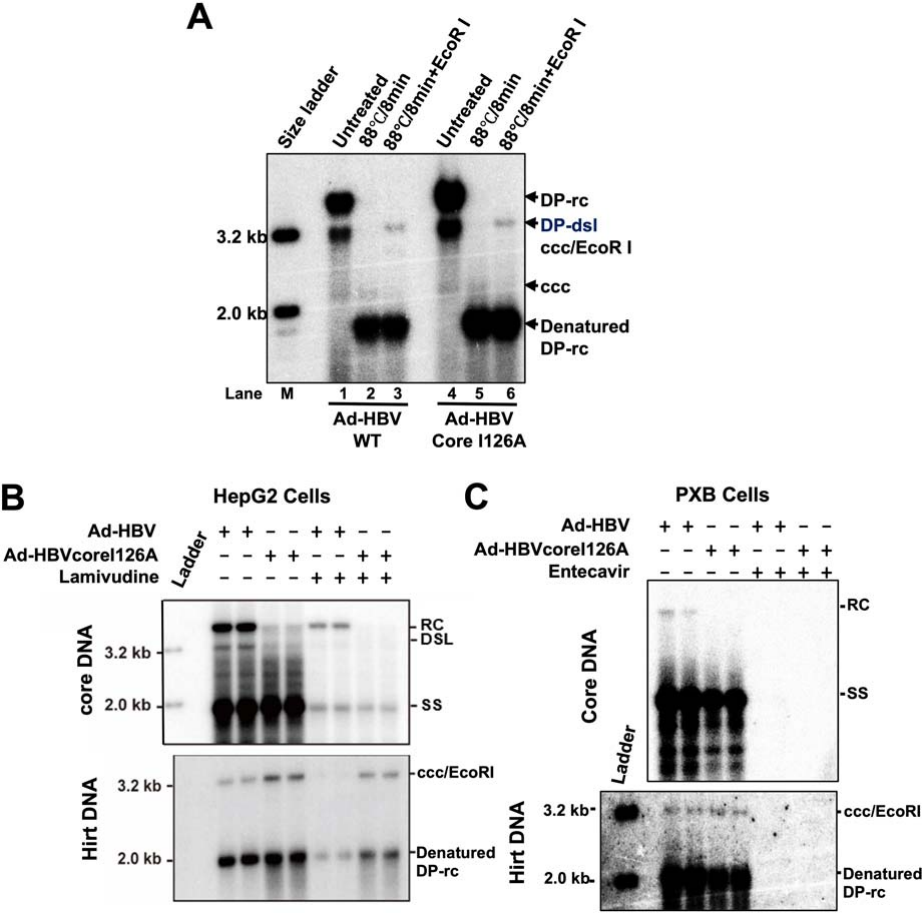
Mature nucleocapsid uncoating supports cccDNA biosynthesis in human hepatocytes. Note: (A) HepG2 cells were infected with Ad-HBV or Ad-HBV-I126A at an MOI of 10 and cultured in the absence of tet. The cells were harvested at day 6 post infection and Hirt DNA was extracted and analysed by Southern blot assay without treatment (lanes 1 and 4), after denaturation at 88°C for 8 min (lanes 2 and 5) or EcoRI digestion after heat denaturing (lanes 3 and 6). (B and C) HepG2 and PXB cells were infected with Ad-HBV or Ad-HBV-I126A at an MOI of 10 and cultured in the absence of tet. The Cells were mock-treated or treated with 10 μM of lamivudine (HepG2) or 1 μM of entecavir (PXB cells) from day 2 to day 6 after infection. HBV core DNA and Hirt DNA were extracted and analysed by Southern blot hybridization. Hirt DNA extracted from the cells were denatured at 88°C for 8 min to denature DP-rcDNA to single-stranded DNA and followed by restriction with EcoRI to convert cccDNA into unit-length double DNA.

To determine whether adenoviral vector-mediated tet-inducible HBV replication in human hepatoma cells supports cccDNA synthesis, Hirt DNA was extracted from HepG2 cells transduced with Ad-HBV or Ad-HBVcoreI126A. As shown in Figure 4(A), three HBV DNA species, supercoiled cccDNA, deproteinized double-stranded linear (DP-dsl) DNA and deproteinized relaxed circular (DP-rc) DNA, can be observed in Hirt DNA preparation (Lanes 1 and 3). As expected, heat denaturing of the Hirt DNA converted DP-dsl and DP-rcDNA into single-strand DNA (lanes 2 and 4), whereas the cccDNA is resistant to heat denaturing and can be digested by EcoRI into unit-length (3.2 kb) linear DNA (lanes 3 and 6). These results clearly indicate that the replication of both wild-type and coreI126A mutant HBV in human hepatoma cells support cccDNA synthesis. To investigate the effect of nucleoside analogue (NUC) antiviral therapy on cccDNA synthesis, Ad-HBV or Ad-HBVcoreI126A infected HepaG2 cells were mock-treated or treated with lamivudine. As shown in Figure 4(B), both wild-type and CpI126A mutant HBV replication support efficient cccDNA synthesis and in agreement with a previous report, CpI126A mutant virus promoted cccDNA intracellular amplification in human hepatoma cells. However, significant (but incomplete) inhibition of HBV DNA replication by lamivudine does not efficiently inhibit cccDNA intracellular amplification in HepG2 cells. This observation implies that a slight leakiness of HBV DNA synthesis suppression under nucleos(t)ide analogue antiviral therapy might be sufficient to maintain the pool of cccDNA in virally infected hepatocytes and result in the failure of cure of HBV infection.

Due to the low copy numbers of HBV cccDNA in HBV infected primary human hepatocytes (PHHs), it had been argued whether the intracellular cccDNA amplification pathway functions in PHHs. To answer this question, human hepatocytes recovered from uPA-SCID mice, i.e. PXB-cells, were infected by Ad-HBV or Ad-HBVcoreI126A and cultured in the absence or presence of HBV DNA polymerase inhibitor entecavir for six days. As shown in Figure 4(C), a full-spectrum of HBV DNA replication intermediates can be found in Ad-HBV infected cells, whereas full-length rcDNA was undetectable in Ad-HBVcoreI126A-infected cells. However, DP-rcDNA and cccDNA can be detected in the cells infected by both the recombinant viruses. Not surprisingly, entecavir treatment completely inhibited HBV DNA replication, and consequentially, cccDNA synthesis did not occur.

### Three-prime repair exonuclease 1 (TREX1) efficiently digests HBV rcDNA and inhibits cccDNA intracellular amplification in human hepatoma cells

In our efforts to investigate the mechanism of cccDNA intracellular amplification, we found that DP-rcDNA (or protein-free DNA) existed in both cytoplasmic and nuclear fractions and the cytoplasmic fraction of DP-rcDNA co-sedimented with nucleocapsids in sucrose gradient centrifugation and were sensitive to DNase I digestion *in vitro* [16,17,25]*.* These findings allowed us to postulate that the completion of plus-strand DNA synthesis may trigger the removal of viral DNA polymerase covalently linked to the 5′-terminus of minus-strand DNA, i.e. dslDNA and rcDNA deproteinization, as well as partial disassembly of mature nucleocapsids in the cytoplasm [16,17,25,26]. The partial disassembly of mature nucleocapsids leads to the exposure of the nuclear localization signal (NLS) located at the carboxyl terminal domain (CTD) of Cp and import of DP-dslDNA and DP-rcDNA into the nuclei for cccDNA synthesis [25]. To rigorously determine the subcellular compartment of progeny mature nucleocapsid disassembly and production of DP-rcDNA, an ideal experimental approach is to test whether the DP-rcDNA is sensitive to a cytoplasmic DNase digestion.

Interestingly, it was demonstrated by several independent laboratories that human immunodeficiency virus (HIV) infection and activation of innate immune response in infected cells are regulated by a cytoplasmic 3′→5′ exonuclease TREX1, which digests both single- and double-stranded DNA [27–30]. While depletion of TREX1 expression in monocytes increased early viral cDNA and enhanced HIV-1 activation of cGAS-STING pathway, increasing the expression of TREX1 almost completely diminished HIV-1 induction of innate cytokine response. However, manipulation of TREX1 expression did not significantly alter the levels of productive infection and integrated proviruses [31]. These observations indicate that while the cytoplasmic uncoated viral DNA that are capable of activating cGAS is TREX1 sensitive, the integration-competent HIV-1 genomes are shielded from cytosolic sensors and nucleases during uncoating and nuclear importation [32].

**Figure 5.**
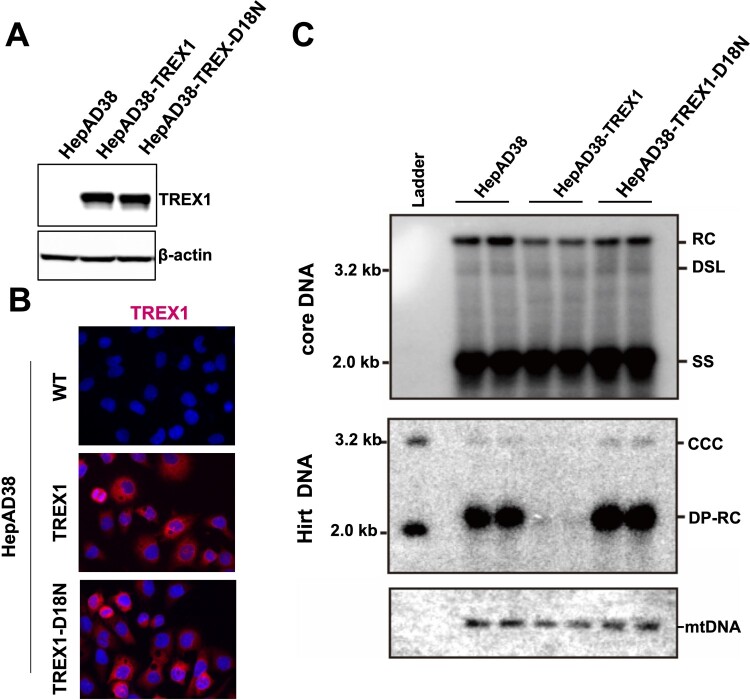
Over-expression of TREX1 selectively digests mature double-stranded HBV DNA in HepAD38 cells. Note: (A) Expression of wild-type and mutant TREX1 in the indicated cell lines was detected by a western blot assay with an antibody against TREX1. *β*-actin served as a loading control. (B) Cytoplasmic localization of wild-type and mutant TREX1 in the indicated cell lines was indicated by an immunofluorescent assay with an antibody against TREX1. (C) Hirt DNA extracted from the indicated cells were denatured at 88°C for 8 min to denature DP-rcDNA to single-stranded DNA and followed by restriction with EcoRI to convert cccDNA into unit-length double stranded linear DNA and detected by Southern blot hybridization. Host cellular mitochondrial DNA served as loading controls for Hirt DNA.

Encouraged by the findings in HIV research, we intended to test the effect of TREX1 expression in human hepatoma cells on DP-rcDNA production and cccDNA synthesis. To this end, we first established a HepAD38-derived cell line that expresses either wild-type TREX1 or exonuclease inactive TREX1 (TREX1-D18N) by recombinant retroviral transduction, designated as HepAD38-TREX1 and HepAD38-TREX1-D18N, respectively [33]. Western blot assay demonstrated that while parental HepAD38 cells express undetectable level of TREX1, expression of TREX1 protein in HepAD38-TREX1 and HepAD38-TREX1-D18N cells was readily detectable (Figure 5(A)). Immunofluorescent staining confirmed that TREX1 was exclusively expressed in the cytoplasm (Figure 5(B)). Interestingly, compared to parental HepAD38 cells, ectopic expression of wild-type TREX1, but not TREX1-D18N, modestly reduced the amount of cytoplasmic HBV rcDNA, but abolished the accumulation of DP-rcDNA and cccDNA (Figure 5(C)). The results from this intracellular nuclease protection assay thus strongly support the hypothesis that the disassembly of progeny mature nucleocapsids and rcDNA deproteinization take place in the cytoplasm and DP-rcDNA is a functional precursor of cccDNA amplification in human hepatocyte-derived cells.

**Figure 6.**
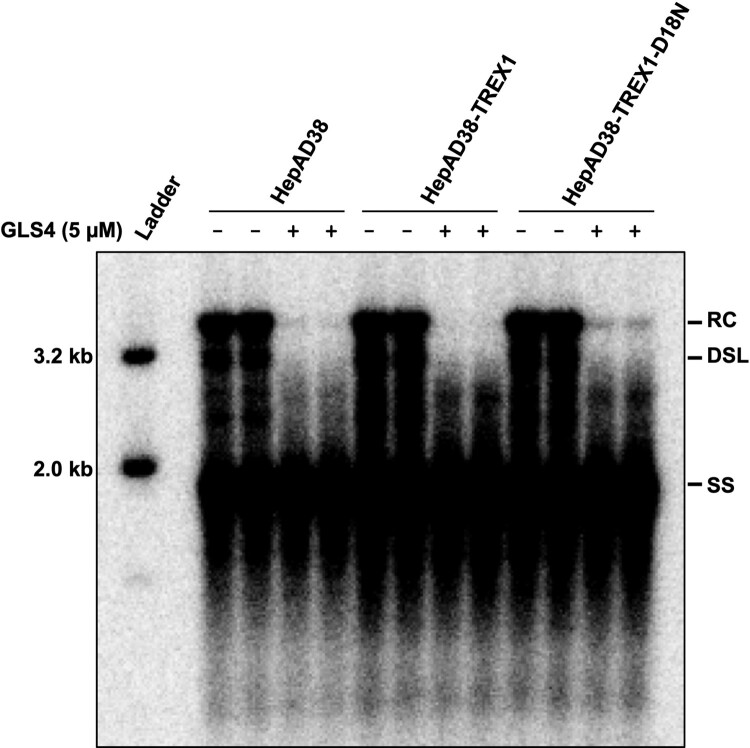
Endogenous TREX1 does not play a significant role in elimination of cytoplasmic double-stranded HBV DNA in GLS4-treated cells. Note: Parental HepAD38 and its derived cell lines were cultured in the absence of tet for 6 days. The cells were then mock-treated or treated with 5 μM of GLS4 for 24 h. Cytoplasmic HBV core DNA were extracted from the cells and determined by Southern Blot hybridization.

Moreover, the sensitivity of uncoated viral DNA to the cytoplasmic nuclease TREX1 and significant reduction of mature forms of HBV DNA species in hepatocytes supporting the replication of HBV expressing CpI126A or under the condition of CpAM treatment suggest that the uncoated viral DNA can be degraded by cytoplasmic nucleases. In order to determine whether cellular endogenous TREX1 plays a role in the degradation of uncoated viral DNA under these conditions, parental HepAD38, HepAD38-TREX1 and HepAD38-TREX1-D18N cells were cultured in the absence of tet for 6 days and then mock-treated or treated with 5 μM of GLS4 for 24 h. As anticipated, GLS4 treatment induced disassembly of mature nucleocapsids and degradation of dsl and rcDNA (Figure 6). In agreement with the undetectable level of endogenous TREX1 expression in HepG2 (Figure 5(A)), over-expression of TREX1-D18N, which can act as a dominant-negative suppressor of endogenous wild-type TREX1 [33,34], only slightly attenuated the reduction of rcDNA in GLS4 treated cells (Figure 6). It is, therefore, conceivable that cytoplasmic nuclease(s) other than TREX1 are responsible for the digestion of uncoated viral DNA and modulation of HBV cccDNA amplification in hepatocytes.

### Expression of TREX1 does not inhibit cccDNA synthesis in *de novo* HBV infection of C3A^hNTCP^ cells

**Figure 7.**
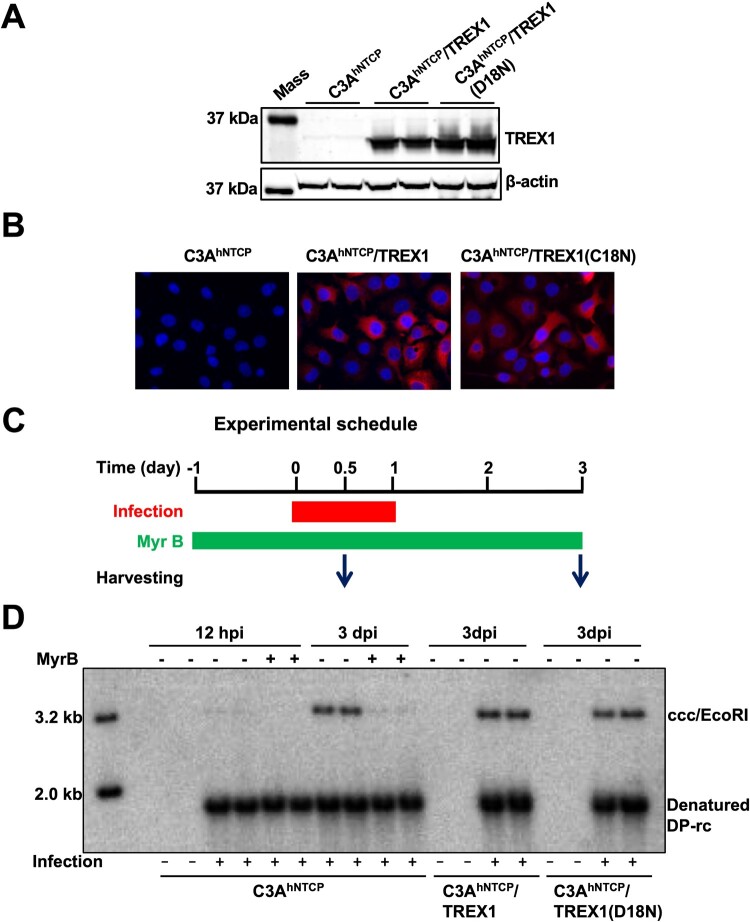
Expression of TREX1 does not inhibit the synthesis of cccDNA in *de novo* infection. Note: (A) Expression of wild-type and mutant TREX1 in the indicated cell lines were detected by a western blot assay with an antibody against TREX1. *β*-actin served as a loading control. (B) Cytoplasmic localization of wild-type and mutant TREX1 in the indicated cell lines were indicated by an immunofluorescent assay with an antibody against TREX1. (C) Schematic presentation of HBV infection and treatment schedule. (D) Hirt DNA were extracted at 12 and 72 h post infection and analysed by Southern blot hybridization.

Our recent studies showed that while CpAM-induced uncoating of cytoplasmic progeny nucleocapsids accelerated cccDNA intracellular amplification, CpAMs inhibited cccDNA synthesis in *de novo* HBV infection [12]. This finding suggests that the cytoplasmic transportation and/or disassembly of incoming virion-derived nucleocapsids might be different from the cytoplasmic progeny mature nucleocapsids. As we have already found that TREX1 can efficiently digest uncoated viral DNA upon the disassembly of cytoplasmic progeny nucleocapsids and inhibit cccDNA intracellular amplification in HepAD38 cells, we examined whether expression of TREX1 modulates cccDNA synthesis during *de novo* HBV infection of C3A^hNTCP^ cells. As shown in Figure 7(A,B), cytoplasmic expression of wild-type and mutant TREX1 in C3A^hNTCP^ cells was confirmed by Western blot and immunofluorescent staining assays. Interestingly, while Myrcludex B, an HBV entry inhibitor [35], completely blocked HBV infection and prevented cccDNA formation, expression of either wild-type TREX1 or TREX1-D18N did not apparently alter the amounts of cccDNA in the infected cells (Figure 7(C)). This finding indicates that, in striking contrast with progeny nucleocapsids that disassemble in the cytoplasm, the uncoated virion rcDNA and synthesized cccDNA are completely protected from the cytoplasmic nucleases in *de novo* HBV infection, suggesting that either the disassembly of incoming virion-derived nucleocapsids does not occur until docking into nuclear pore complex, or the uncoated viral DNA is protected from cytoplasmic nuclease digestion *via* unknown mechanisms.

## Discussion

Nucleocapsid, a structural entity of viral genome coated with a protein shell, is a vehicle that transports viral genome among susceptible cells and individuals. The unique function of nucleocapsids requires them to be assembled in infected cells and disassemble upon infection of susceptible cells so as to release viral genome (i.e. uncoat) and initiate viral replication. The ability of HBV mature progeny nucleocapsids to disassemble without leaving the host cells where they get assembled is a very unique property of hepadnaviruses and implies that the intracellular disassembly of progeny mature nucleocapsids must be tightly controlled to avoid inappropriate amplification of cccDNA pool, which can be detrimental to the infected hepatocytes [36]. Moreover, disassembly of mature nucleocapsids not only releases viral genomic DNA for cccDNA synthesis, but also has a potential to activate host cellular DNA sensors and induce an antiviral cytokine response [27,37]. Taking the advantage of CpAM-induced or CpI126A-enhanced uncoating of HBV double-stranded DNA in the cytoplasm [11–13], we found in this study that although transfection of HBV core DNA can efficiently activate the cGAS-STING pathway (Figure 2), enhanced viral double-stranded DNA uncoating failed to do so (Figure 3). These results suggest that either the uncoated viral DNA is not accessible to cGAS or quickly degraded by cytoplasmic nucleases. The undetectable levels of TREX1 expression in hepatoma cells and efficient reduction of uncoated viral double-stranded DNA in hepatoma cells under the conditions of enhanced nucleocapsid disassembly by GLS4 treatment or replication of CpI126A mutant virus imply that one or multiple unknown cellular nucleases can digest uncoated viral DNA in the cytoplasm. It can be speculated that these cytoplasmic nucleases may play an important role in regulating intracellular cccDNA amplification and/or HBV activation of innate immune response in infected hepatocytes. Hence, identification of the responsible nucleases will reveal host cellular factors that modulate HBV infection and pathogenesis.

The sensitivity of DP-rcDNA to TREX1 in HepAD38 cells (Figure 5) supports the hypothesis that the disassembly of progeny mature nucleocapsids and rcDNA deproteinization take place in the cytoplasm and proves that the DP-rcDNA is indeed the functional precursor of cccDNA synthesis [16,17,25]. However, the complete resistance of cccDNA synthesis to TREX1 in *de novo* HBV infection of C3A^hNTCP^ cells (Figure 7) suggests that the disassembly of nucleocapsids under those conditions may take place in the cellular compartments without high concentrations of TREX1, such as at nuclear pore complexes (NPCs) or in the nuclei. Alternatively, it is also possible that the uncoated viral DNA is protected by host cellular factors in the cytoplasm to escape from TREX1 digestion. Further studies are required to distinguish these possibilities.

**Figure 8.**
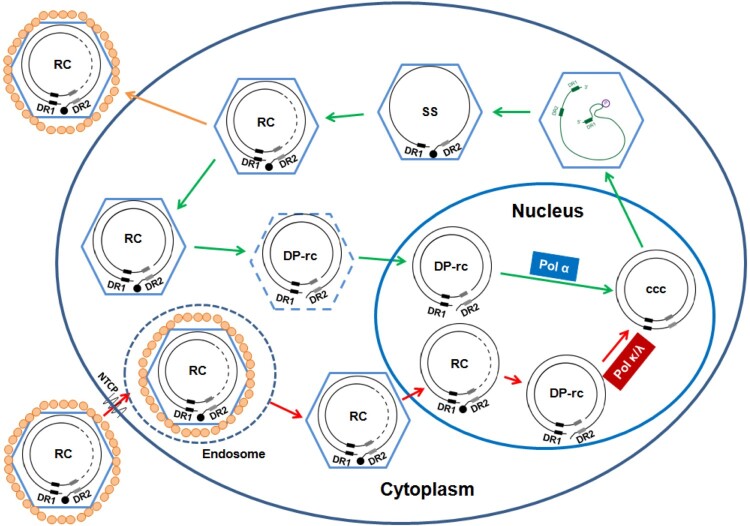
Schematic illustration of the difference in *de novo* cccDNA synthesis and intracellular cccDNA amplification pathways. Note: The nucleocapsid with dashed line indicates partially disassembled nucleocapsid. The DP-rcDNA associated with the partially disassembled nucleocapsid is sensitive to nuclease digestion, but can be imported into the nucleus for cccDNA synthesis. DR1 and DR2 indicate the direct repeat DNA sequence 1 and 2, respectively. The black filled dot at the 5′ end of minus strand DNA represents viral DNA polymerase. See text in introduction section for detailed explanation of viral replication cycle.

Interestingly, in addition to the distinct mechanisms of nucleocapsid uncoating in the two cccDNA synthesis pathways, we recently found that while *de novo* cccDNA synthesis requires cellular DNA polymerase κ and λ [38], intracellular amplification of cccDNA relies on DNA polymerases α [39] (illustrated in Figure 8). The distinct usage of host DNA polymerases, and possibly other DNA repair proteins, by the two cccDNA synthesis pathways might be due to their precursors, the rcDNA, are differentially uncoated and imported into the distinct subdomains of the nucleus. It can be anticipated that unravelling the mechanism and regulation of HBV cccDNA synthesis will reveal molecular targets for rational development of novel therapeutics to purge the pool of cccDNA or activate innate immune response in infected hepatocytes and ultimate cure chronic HBV infection.
